# Benefits of fading in perceptual learning are driven by more than dimensional attention

**DOI:** 10.1371/journal.pone.0180959

**Published:** 2017-07-19

**Authors:** Matthew G. Wisniewski, Milen L. Radell, Barbara A. Church, Eduardo Mercado

**Affiliations:** 1 711^th^ Human Performance Wing, U.S. Air Force Research Laboratory, Wright-Patterson Air Force Base, Ohio, United States of America; 2 Department of Psychology, Niagara University, Lewiston, New York, United States of America; 3 Department of Psychology, Language Research Center, Georgia State University, Georgia, United States of America; 4 Department of Psychology, University at Buffalo, The State University of New York, Buffalo, New York, United States of America; Universitat Zurich, SWITZERLAND

## Abstract

Individuals learn to classify percepts effectively when the task is initially easy and then gradually increases in difficulty. Some suggest that this is because easy-to-discriminate events help learners focus attention on discrimination-relevant dimensions. Here, we tested whether such attentional-spotlighting accounts are sufficient to explain easy-to-hard effects in auditory perceptual learning. In two experiments, participants were trained to discriminate periodic, frequency-modulated (FM) tones in two separate frequency ranges (300–600 Hz or 3000–6000 Hz). In one frequency range, sounds gradually increased in similarity as training progressed. In the other, stimulus similarity was constant throughout training. After training, participants showed better performance in their progressively trained frequency range, even though the discrimination-relevant dimension across ranges was the same. Learning theories that posit experience-dependent changes in stimulus representations and/or the strengthening of associations with differential responses, predict the observed specificity of easy-to-hard effects, whereas attentional-spotlighting theories do not. Calibrating the difficulty and temporal sequencing of training experiences to support more incremental representation-based learning can enhance the effectiveness of practice beyond any benefits gained from explicitly highlighting relevant dimensions.

## Introduction

Two perceptual events that are difficult or impossible for an individual to distinguish can become discriminable through a training procedure that starts with easy distinctions and gradually progresses to more subtle differences [1–2]. This phenomenon has been referred to as the *easy-to-hard effect* or *transfer along a continuum*, while the progressive procedures used to induce the effect have been termed *fading* or *progressive training*. Pavlov [2] demonstrated the easy-to-hard effect in dogs learning to discriminate visual, auditory, and somatosensory stimuli. Early studies in humans also showed easy-to-hard effects for simple images and sounds [3–5]. More recent work has established that fading influences not only acquisition, but also perceptual generalization [6–7] and cortical plasticity [8]. The easy-to-hard effect was once a major focus of associative learning research because of extensive debates about whether effects were due to an increase in dimensional salience [9], or acquired gradients of association (reviewed by [10]). Recently, similar debates have arisen in the context of perceptual learning/perceptual category learning studies, with some researchers again arguing that this effect is due to increases in dimensional salience [11–12], whereas others argue that the effect can be explained in terms of gradual changes in stimulus representations and/or their associations [13–20].

Those arguing for the dimensional salience perspective posit that progressive training serves to highlight relevant dimensions. Imagine that a listener is given the task of discriminating two similar tones of 1000 and 1005 Hz. If not told that the relevant dimension is frequency, the listener will need to discover this on their own to successfully distinguish the sounds, for example, by ruling out other possible dimensions such as sound duration or intensity. Learning may be slowed by tests of various hypotheses regarding the relevant dimension. In contrast, if initial experimental trials present an easily discriminable difference (e.g., 1000 vs. 1300 Hz), the listener will become immediately aware that frequency is relevant to performing the task. This then facilitates discrimination of smaller frequency differences. In essence, this attentional-spotlighting perspective argues that progressive training causes an attention-related “stretching” of a dimension by facilitating the discovery that the dimension is relevant. This idea has been pervasive in the perceptual learning [21–23] and category learning literatures [11, 24–26]. Several popular learning theories/models (e.g., ALCOVE; [27]; Analyzer Theory; [28]) have incorporated such mechanisms.

In contrast, associative theorists suggest that the associations between stimulus representations and behaviorally relevant outcomes constrain discrimination performance (for reviews see [16–17, 29–30]). While learning highly discriminable events, there is little overlap between stimulus representations. Learning in this scenario is fast and favors elements of representations that are active on a particular trial—most of which are unique to each stimulus. When more difficult discriminations are introduced, the elements that help distinguish these stimuli will already carry the most associative strength, thus facilitating learning. If an individual only experiences difficult discriminations, the most active elements may be those that are shared by both stimuli. Learning will then proceed more slowly [14,16,20]. There are similar theories that employ physiologically plausible stimulus representations (e.g., activations of artificial visual cortical neurons with fixed response profiles) as inputs to associative learning-based artificial neural networks [31]. Researchers employing these models have posited that associative weights represent weights of attention [32]. However, this process of incremental attentional shift involves differentially weighting portions of dimensions/representations, making it a different learning process than dimensional discovery.

Still another class of representation-based learning theories explain the easy-to-hard effect with mechanisms of non-associative learning that involve gradual, experience-dependent changes to stimulus representations themselves. For example, Saksida [18–19] developed a neural network model composed of a self-organizing map (SOM) competitive learning layer, and an associative output layer responsible for mapping representations in the SOM to response categories. A key feature of the model was that competitive learning in the SOM enabled internal representations of stimuli to change over the course of exposure (see [33]). When trained with only difficult-to-discriminate inputs, stimuli continually competed over the same representational space in the map (i.e., they activated the same processing elements). Consequently, representational modification was slow, degrading the ability of the associative learning layer to map stimuli to correct outputs. In contrast, when the model was initially trained with easy contrasts, competition for representational space between hard-to-discriminate stimuli was reduced. This was because the SOM had already spatially segregated elements that distinguished inputs. In Saksida’s model [18–19], easy trials facilitated later representational modification, making it easier to map similar stimuli to different outputs. Importantly, the SOM computational framework (also, [33–34]) predicts learning and the easy-to-hard effect regardless of whether or not attention is directed toward particular stimulus features. This prediction is consistent with neural data demonstrating that under passive exposure conditions there is significant reorganization of cortical representations of stimuli (e.g., [35–36]) and improvements in perceptual performance (e.g., [6, 37–40]). It can also potentially account for learning along stimulus dimensions that are irrelevant for making trained discriminations (e.g., [41–45]) and the specificity of learning within a dimension (for review, see [46]).

Whether the advantages of progressive training arise from the discovery of appropriate dimensions, from adjustments to stimulus representations and their outputs, or from both processes, continues to be debated. Suret and McLaren [47] created four different morphed face continua and trained participants in a categorization task with an easy-to-hard or constantly difficult regimen. Despite training on the four continua concurrently (i.e., several dimensions were relevant in the same task), easy-to-hard effects were still found. Those results were well simulated by a simple associative model of learning, leading the authors to conclude that “there is no need in our theorizing to postulate changes in associability to a dimension as a whole”. In a series of recent studies from our group [6–8], progressively trained human listeners outperformed those receiving non-progressive training in auditory temporal discrimination tasks. In one study, we used birdsongs that varied in overall rate as stimuli [6]. Training regimens in which participants progressed from easy-to-hard discriminations, moved from hard-to-easy discriminations (anti-progressive), had randomly ordered discrimination difficulties, or constantly hard discriminations were compared. Progressively trained participants showed the best discrimination performance. Participants undergoing anti-progressive training performed the worst. This suggested that it was the progression from easy-to-hard discriminations, and not mere variability in discrimination difficulty that mattered. Also of note, even though participants in the anti-progressive and random-order regimens received easy trials that emphasized temporal dimensions of difference, this did not lead to performance equal to that of progressive training.

More support for representation-based views comes from neurophysiological work. One study in which barn owls were exposed to prismatic spectacles, found that receptive fields of neurons coding for auditory space in the optic tectum were altered more so when prisms shifted the horizontal visual field in progressive increments than when a large shift was introduced without progression [48]. Another study investigated whether differences in human performance were correlated with differences in cortical plasticity observable in the auditory-evoked potential (AEP). Participants were trained in either a progressive or constantly difficult regimen to discriminate frequency modulated sounds with a 12 Hz repetition rate from sounds having slower rates (i.e., <12 Hz). AEPs were measured before and after training in a paradigm in which frequently presented 12 Hz sounds were intermixed with occasional oddball sounds with slower rates. The P2 component of the AEP evoked by slow sounds showed amplitude enhancement that was greater after progressive training. This effect was obtained while participants were asked to ignore sounds and read a book or magazine, suggesting that neural signatures of the progressive advantage are observable under conditions in which attention is not directed toward stimuli [8].

Drawing contrasting conclusions, Pashler and Mozer [11] reported that the benefits of progression in perceptual category learning tasks were only evident when the relevant stimulus dimension was obscured by varying features along multiple dimensions. Furthermore, when Pashler and Mozer’s participants were explicitly informed of the relevant dimension prior to training, the progressive advantage disappeared. Based on these results, they suggested that progression enhances learning primarily in situations where participants are confused about what features distinguish the events to be classified. Similar results and conclusions were reached decades earlier in other category learning experiments [2,3,5,9]. Similarly, Ahissar and Hochstein [49] found that exposure to a single “target present” and “target absent” trial in an easy version of a visual detection task was sufficient to facilitate later learning. Few participants showed learning without easy trial exposures. This *Eureka effect* was interpreted to support the idea that easy trials make stimulus features accessible via attention. There is further support for this view in observations that having knowledge regarding an upcoming trial’s difficulty or relevant perceptual dimension facilitates stimulus processing as assessed with behavioral [50–51] and physiological measures [52].

Understanding the mechanisms that drive easy-to-hard effects, and testing assumptions of current learning theory, are important for the development of perceptual training regimens that have real-world applications (e.g., speech contrast training, dialect accommodation training, bird identification, etc.). If easy-to-hard effects are mainly driven by the discovery of the dimensions that contain the most information, then training regimens may be most effective if they explicitly focus attention on relevant dimensions. For instance, Roads, Mozer, & Busey [53] propose that lengthy visual expertise training in fingerprint-matching can be reduced if novices are guided where to look by increasing the saliency of the parts of a fingerprint image where experts look. Similar proposals have been made for learning of unfamiliar speech sound contrasts [24]. If, however, data suggest that incremental representation-based learning mechanisms are an important component of refining perceptual abilities, then training regimens may be more effective when they take into account how sequencing of stimulus presentations constrains learning-related changes to stimulus representations and/or their associations.

The current work tests the popular claim that attentional spotlighting views are sufficient to explain easy-to-hard effects in perceptual/category learning. If benefits are a result of discovering the appropriate dimension (cf. [11]), then any benefit of sequencing should generalize across the critical dimension. For example, if easy-to-hard sequencing causes the attentional spotlight to be placed on the relevant auditory dimension of frequency modulation (FM) rate (cf. [6–8]), benefits should apply to all stimulus contrasts in which the critical dimension is FM rate. Several researchers have used this exact argument in support of attentional-spotlighting in categorization tasks (e.g., [27]). In contrast, learning theories based on how representations are reorganized and modified predict that benefits should be partially specific to the feature values of trained stimuli. For instance, if learning is enhanced under a regimen that fades from large to small FM rate differences, this learning will be specific to those rates, and potentially the audible sound frequencies present in the acoustic signal [46]. In other words, the progressive advantage should be restricted to the stimulus representations elicited by progressively trained stimuli. This work tested these hypotheses via within-subjects designs where participants received easy-to-hard sequencing of FM rate discriminations for trains of FM sweeps in one frequency range (e.g., 300–600 Hz), but constantly difficult discriminations for FM sweep trains that spanned a separate set of frequencies (e.g., 3000–6000 Hz). If learning mechanisms beyond attentional-spotlighting contribute to the benefits associated with progression, then participants should perform better after training with sounds from their progressively trained range. This is examined both under testing conditions very similar to training (Experiment 1), and testing conditions requiring application of learning to a novel and more difficult task (Experiment 2).

## Experiment 1

In Experiment 1, participants were trained simultaneously in progressive and constantly difficult training regimens to categorize FM sweep trains with different rates of frequency modulation as ‘Fast’ or ‘Slow’. Progressive and Constant regimens were assigned to different frequency ranges and were counterbalanced. Participants were tested post-training with both frequency ranges. Effects of regimen were examined.

## Methods

### Ethics statement

The Institutional Review Board of The University at Buffalo, State University of New York, approved Experiment 1 of this study. All participants signed an informed consent document.

### Participants

Twenty-two young adults (ages 18–32) from the area surrounding the University at Buffalo, The State University of New York, participated in exchange for course credit in an introductory psychology course, or on a volunteer basis. Two participants, one from each group, were excluded from the analysis because they failed to exceed chance performance in the testing portion of the experiment (averaged across frequency ranges). All participants were putatively healthy with self-reported normal hearing. Participants were assigned randomly to either receive progressive training in the ‘low’ or the ‘high’ frequency range, with constant training assigned to the opposite range.

### Stimuli and apparatus

**Fig 1:**
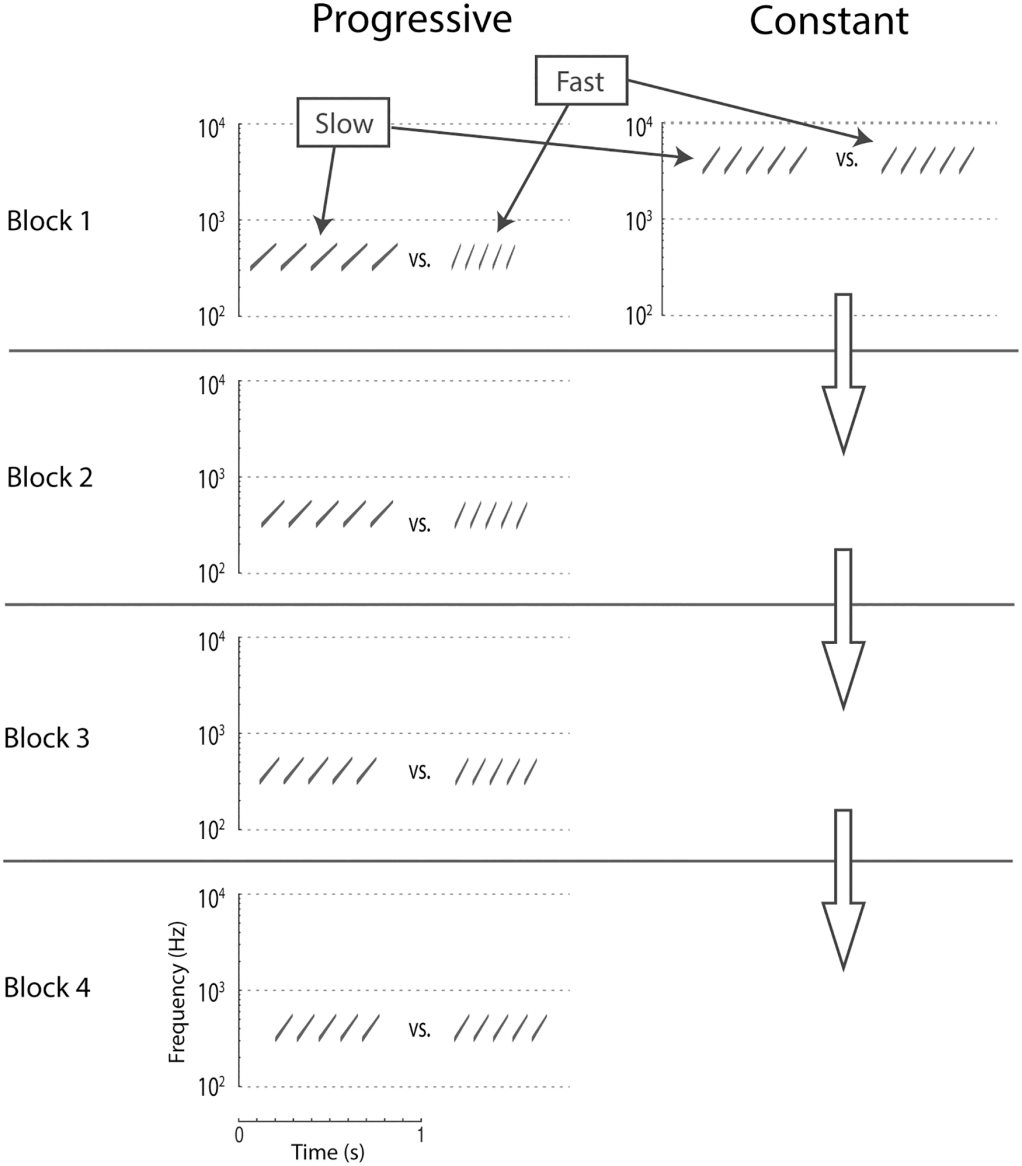
Spectrograms of sweep trains and depiction of training procedures for the counterbalance condition which received progressive training in the ‘low’ frequency range and constant training in the ‘high’ frequency range. In the low frequency range, Fast/Slow contrasts start with a large difference in Block 1 of training, but progressively become more difficult. In the high frequency range, contrasts start difficult to differentiate and remain difficult throughout training.

Sweep trains consisted of 5 consecutive and upwardly directed FM sweeps spanning frequencies from 300–600 Hz (‘low’ frequency range) or 3000–6000 Hz (‘high’ frequency range). FM rates of 6, 6.7, 7.5, 8.4, 9.4, 10.6, 11.8, and 13.4 octaves per second were used. A preliminary experiment revealed that FM sweep trains in the ‘high’ and ‘low’ frequency ranges were similarly discriminable on the dimension of rate (see supplemental materials, S1 File). See Fig 1 for depictions of example sweep trains. FM sweep trains are especially suitable for the current study because: 1) perceptual learning has been well documented with FM stimuli [7,24,45,54], 2) FM sweep trains are complex, like many real-world sounds (e.g., speech), yet are unfamiliar to participants, and 3) auditory perceptual learning is in many instances frequency dependent, showing partial to full specificity to the frequencies of trained sounds (for review, see [46]). The last reason warrants within-subject comparisons between ‘low’ and ‘high’ frequency ranges after training (cf. [45]). Stimuli were generated in MATLAB 2014a (Mathworks, Natick, MA).

Experimental procedures and data acquisition were performed using DMDX experimental software [55]. Participants made responses via a computer keyboard. Sounds were presented over closed JVC HA-RX500 headphones in closed room judged as quiet by the experimenters. Stimuli were presented at a fixed comfortable listening level not exceeding 81 dB SPL.

### Procedures

Training and testing took place in a single session.

#### Training

A single-interval two-alternative forced choice (1i-2afc) task was used. On half of all trials, a “Slow” sweep train (< = 8.4 octaves per second) was presented. The other half of trials contained “Fast” sweep trains (> = 9.4 octaves per second). Participants were explicitly informed with verbal instructions before starting that the sounds would differ in speed. Their task was to press a key marked ‘S’ if a sweep train was “Slow” and a key marked ‘F’ if a sweep train was “Fast”. Displayed on the screen during each trial was the question: “Slow or Fast?”

Fig 1 depicts training contrasts experienced by half of the participants for which the low frequency range received progressive training and the high frequency range received constant training. In this case, the first block of training contained easy to categorize trains in the ‘low’ frequency range, but hard to categorize trains in the ‘high’ frequency range. Though the contrasts differed in difficulty, the appropriate categorization boundary was the same for these frequency ranges (between 8.4 and 9.4 octaves per second). Over the course of blocks the ‘low’ frequency range included progressively more difficult contrasts until reaching the same FM rate contrast as the ‘high’ frequency range. The ‘high’ frequency range remained at a fixed level of difficulty throughout training. There were 4 blocks of training with 48 trials in each block (12 slow-low, 12 slow-high, 12 fast-low, 12 fast-high). Order of stimuli was pseudo-randomized within a block such that no more than 2 of the same trial types occurred in consecutive trials (unique for each participant). ‘Low’ and ‘high’ frequency range trials were intermixed. Feedback of correctness was given after each trial with the words “Correct” or “Wrong” presented after a response. If a response was not given within 5 s of a sound’s onset, a missing response was recorded and the next trial was initiated. Trials with missing responses were excluded from the analysis. The mean number of missing responses per participant was less than .1% of all trials (maximum was ~2%).

#### Testing

After training, all participants completed a test containing high- and low-frequency range sweep trains at the hardest contrast (8.4 vs. 9.4 octaves per second). The same 1i-2afc task used during training was also used during testing. The test was 84 trials long (21 slow-low, 21 slow-high, 21 fast-low, 21 fast-high). Trial order was pseudo-randomized so that no more than 3 of the same sounds were presented consecutively. No feedback was given during the test.

## Results

### Training

**Fig 2:**
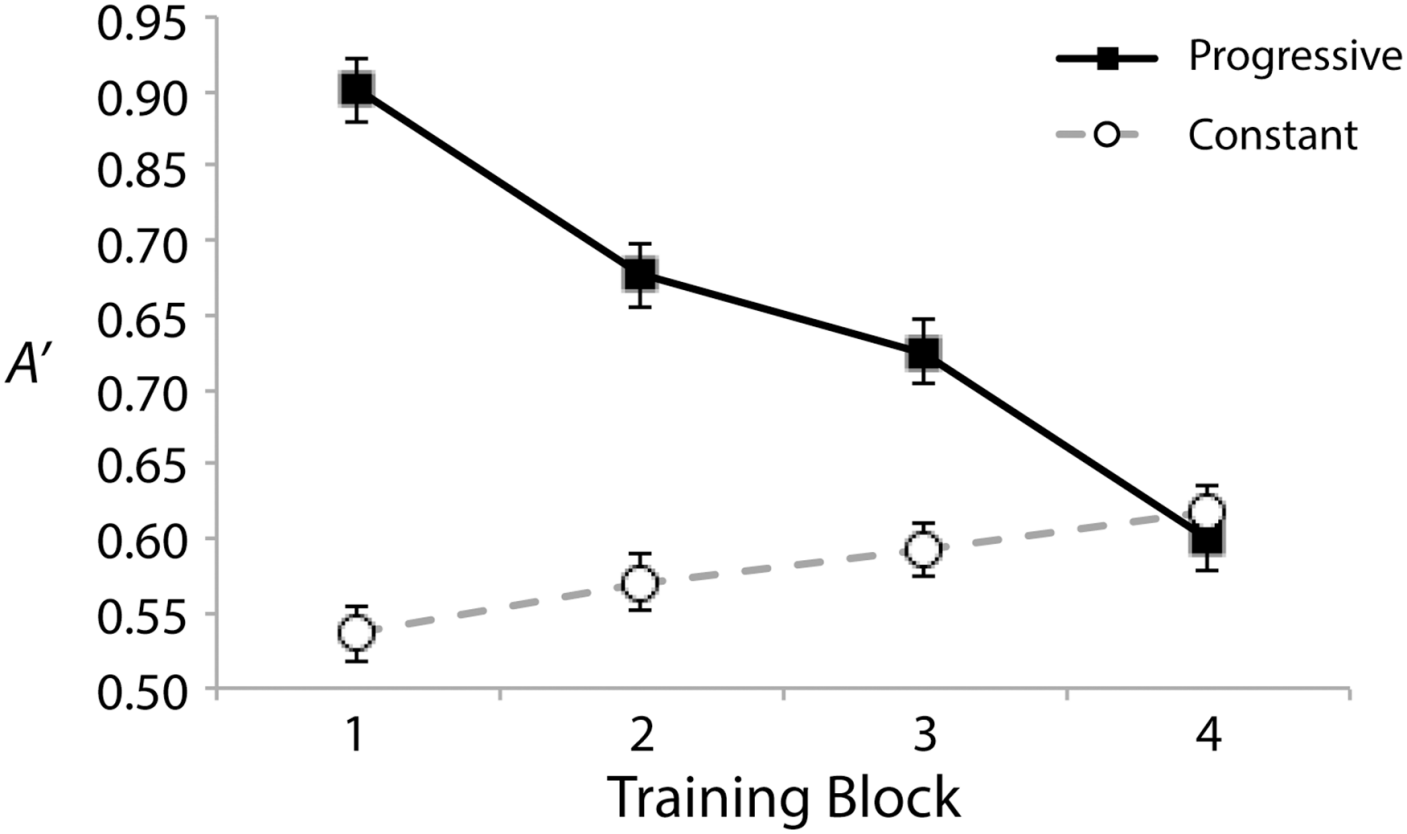
Training performance (*A’*) for the progressive and constant trained frequency ranges. Error bars show within-subject standard errors of the means [57].

Fig 2 shows *A’* in the training portion of the experiment. Data is collapsed across counterbalance conditions, showing performance for the progressively-trained and constant-trained frequency ranges. No significant main effects or interactions with counterbalance condition were found when including it as a factor in any analysis (see S1 File for this alternative analysis of data from both experiments). Where ‘H’ refers to Hit Rate and ‘F’ refers to False Alarm Rate, *A’* was equal to .5 + (H − F)(1 + H − F)/4H(1 − F) when H ≥ F, and .5 − (F − H)(1 + F − H)/4F(1 − H) when H < F [56]. A 2 (range: progressive or constant) x 4 (block) repeated-measures ANOVA revealed both a significant main effect of range, *F*(1, 19) = 46.26, *p* < .001, *η*_*p*_^*2*^ = .71, and of block, *F*(3, 57) = 5.47, *p* = .002, *η*_*p*_^*2*^ = .22. The former reflects the progressive training regimen being easier overall than the constantly hard training regimen. The latter characterizes a large decrease in sensitivity over the course of training that is driven by the large changes in stimulus contrast difficulty from block to block in the progressive condition. There was also a significant range x block interaction, *F*(3, 57) = 20.28, p < .001, *η*_*p*_^*2*^ = .52, stemming from *A’* for the two ranges converging over the course of the training period. This conversion is likely driven by the difficulty of stimulus contrasts becoming more similar between the conditions as training progresses. None of these training results are surprising. They are consistent with previously reported trends between the two types of conditions (e.g., [11]), and are well in line with effects of contrast difficulty on perceptual sensitivity to differences [56]. A noteworthy point, however, is that the block 4 sensitivities appear to be similar for the progressively trained and constant trained ranges. At the end of training, there is no effect of training regimen. Even so, it has been shown that the benefit of progression tends to build up over the course of exposures to a new hard contrast [6, 19]. Test results are better suited for an assessment of training effects.

### Testing

**Fig 3:**
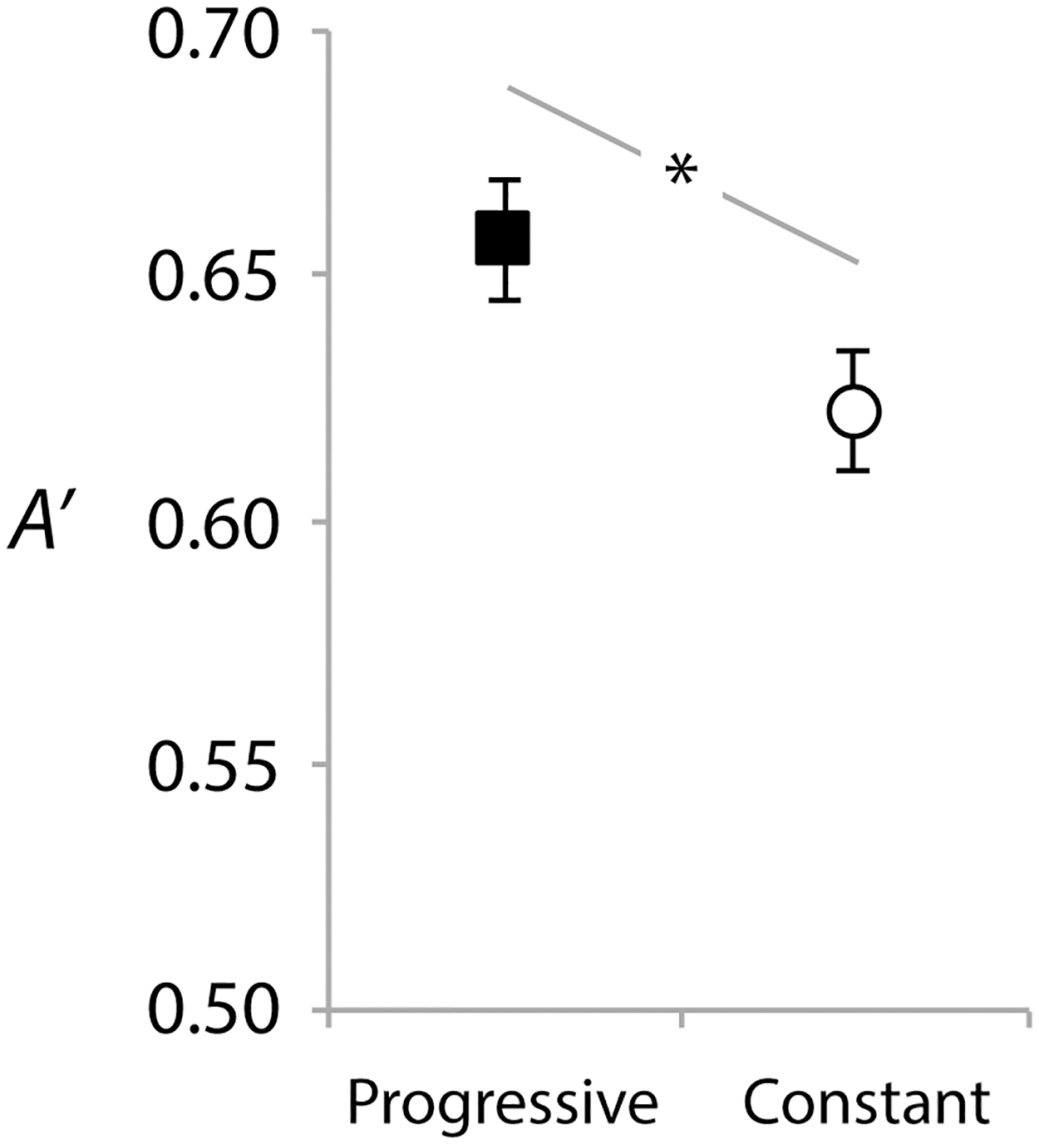
Test performance (*A’*) for the progressive and constant trained frequency ranges. Error bars show within-subject standard errors of the means [57].

Fig 3 shows *A’* in the test for the progressively-trained and constant-trained frequency ranges. A paired samples t-test found that the progressively trained range was performed with significantly higher accuracy than the constant range, *t*(19) = 2.16, *p* = .043, *Cohen’s d* = .49.

## Discussion

That a progressive advantage was found within-subjects when comparing conditions that had the same critical relevant dimension suggests that learning involved processes beyond dimensional-highlighting. Representation-based theories where learning occurs because of changes to the stimulus representations themselves, or in the read-out connections from those representations, predict that benefits should be restricted at least partially to the trained sounds (i.e., the sounds that elicit those representations). Representation-based accounts of perceptual learning are thus more consistent with the current data.

## Experiment 2

Experiment 2 investigated whether or not there is a progressive advantage in the generalization of learning [6–8]. Two types of generalization were examined. First we examined whether the effect would remain when participants were tested in an untrained task. After training similar to Experiment 1, participants were tested in a two-interval two-alternative forced-choice (2i-2afc) task on their ability to discriminate rate in both the progressive and constant trained frequency ranges. Two sounds of differing FM rates were presented, and participants were asked to indicate which was faster. We also tested whether or not there would be a progressive advantage when stimulus contrasts were made more difficult than training. Here, we shortened sweep trains in order to make the task more difficult. Shorter novel sweep trains with less repetitions were tested in addition to trained sweep trains to characterize potential differences in generalization of learning to discriminate more difficult contrasts. There were two reasons for the methodological changes from Experiment 1 to Experiment 2. If the progressive advantage found in Experiment 1 extends to an untrained task (i.e., the 2i-2AFC task) and to untrained stimulus contrasts (i.e., shorter sweep trains), this would provide support for a representation-based account of the progressive advantage. It would also suggest that these perceptual learning mechanisms are potentially relevant for real world training applications because they generalize beyond the exact circumstances of training.

## Methods

### Ethics statement

The Institutional Review Board of United States Air Force Research Laboratory approved Experiment 2 of this study. All participants signed an informed consent document.

### Participants

Eighteen young adults (ages 19–34) at the U.S. Air Force Research Laboratory, Wright-Patterson Air Force Base, OH, were either paid to participate, or served as unpaid volunteers. All individuals had prior experience participating in psychoacoustic studies, including participation in a preliminary study designed to determine whether or not performances in ‘low’ and ‘high’ frequency ranges were comparable (see supplemental materials, S1 File). All participants were putatively healthy with self-reported normal hearing and no psychoactive medication use at the start of the study. Participants were randomly assigned to either receive progressive training in the ‘low’ or the ‘high’ frequency range, with constant training assigned to the opposite range.

### Stimuli and apparatus

Sweep trains were made up of 1, 2, 3, or 4 consecutive and upwardly directed FM sweeps spanning the same frequency ranges as Experiment 1. FM rates of 5, 5.4, 5.8, 6.3, 6.8, 7.4 7.9, and 8.6 octaves per second were used. The number of repetitions and differences between successive FM rates in this stimulus set were reduced to accommodate well-practiced and highly motivated Air Force listeners. As in Experiment 1, stimuli were generated in MATLAB 2014a (Mathworks, Natick, MA).

Experimental procedures and data acquisition were performed using MATLAB. Participants made responses via a computer keyboard. Sounds were presented over Telephonics TDH-39P headphones (Farmingdale, NY) in an Acoustic Systems sound booth (Occupational Health Dynamics, Hoover, AL), at a fixed comfortable listening level not exceeding 81 dB SPL.

### Procedures

The experiment took place across two sessions occurring on separate consecutive days. Training took place in the first session. Testing took place in the second session.

#### Training

The training task and procedures were similar to Experiment 1. On half of the trials, a “Slow” sweep train (< = 6.3 octaves per second) was presented. The other half contained “Fast” sweep trains (> = 6.8 octaves per second). As in Experiment 1, participants received explicit verbal instructions that the sounds would differ in speed prior to starting training. There were 6 blocks of training with 48 trials in each block (12 slow-low, 12 slow-high, 12 fast-low, 12 fast-high). For the progressively trained frequency range, FM rate contrasts faded from easy-to-hard over the course of training blocks: 5 vs. 8.6 (block 1), 5.4 vs. 7.9 (block 2), 5.8 vs. 7.4 (block 3), and 6.3 vs 6.8 (blocks 4–6). The FM rate contrast was always 6.3 vs 6.8 for the constant trained frequency range. Trial order was completely randomized within a block (unique for each participant). Feedback was given in the same manner as Experiment 1. There was no response deadline.

#### Testing

On each test trial two FM sweep stimuli were presented back-to-back with 500 ms of silence in between. One of these stimuli was “Fast” (6.8 octaves per second) and the other was “Slow” (6.3 octaves per second). Listeners’ task was to indicate which was faster. The number of repetitions in FM sweep stimuli (1–4) varied from trial to trial, but was the same for the two stimuli presented within a trial. There were 4 blocks in the test with 48 trials in a block (6 trials for each combination of repetition and frequency range). Orders of the fast and slow sounds within a trial were counterbalanced. Trials were completely randomized within a block (unique for each participant). No feedback was presented. There was no response deadline. Once again, the difference in the testing task serves to examine task generalization of the easy-to-hard effect.

## Results

### Training

**Fig 4:**
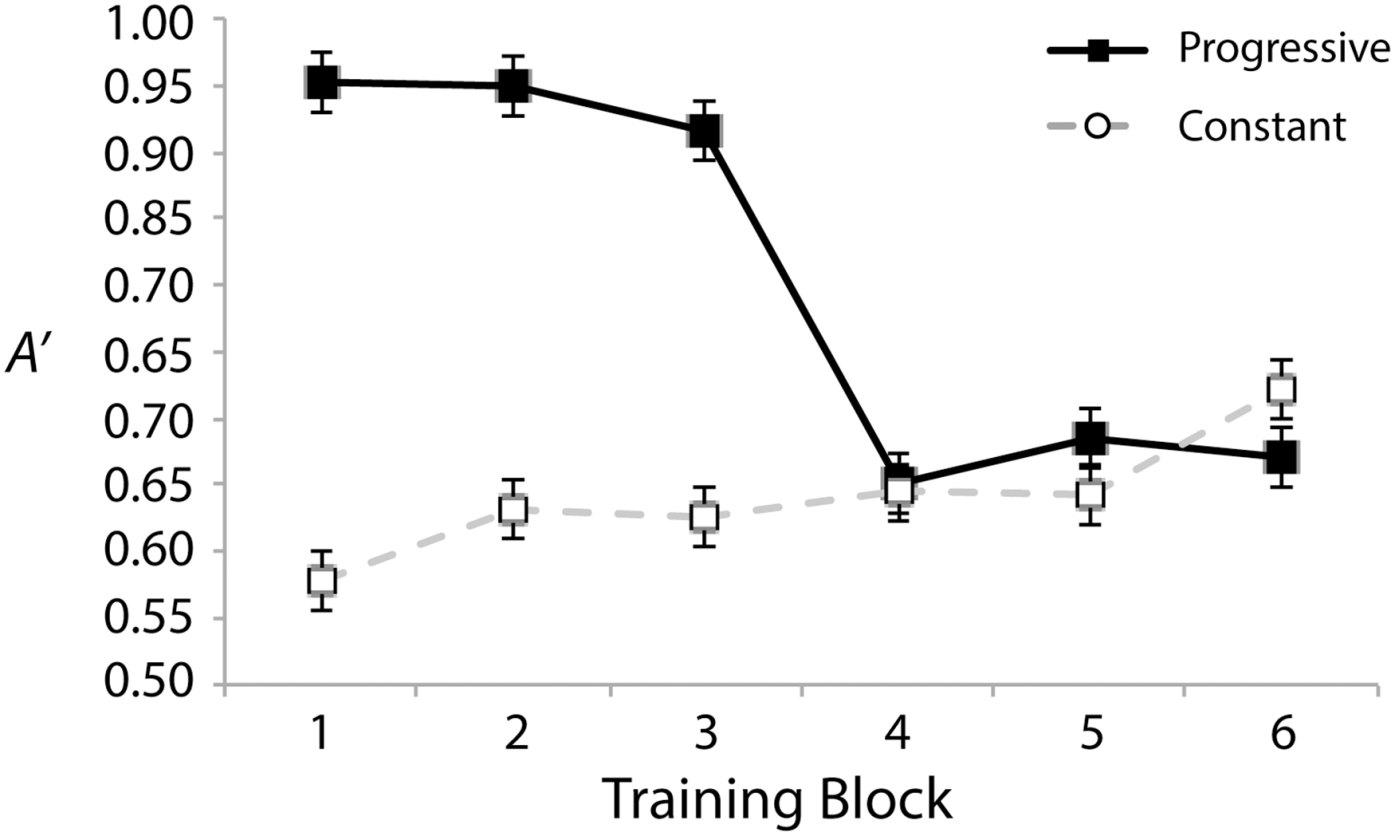
Training performance (*A’*) for the progressive and constant trained frequency ranges. Error bars show within-subject standard errors of the means [57].

As in Experiment 1, all analyses were performed on data collapsed across counterbalance conditions. Fig 4 shows *A’* across training blocks and ranges. A 2 (range: progressive or constant) x 6 (block) repeated-measures ANOVA revealed both a significant main effect of range, *F*(1, 17) = 109.75, *p* < .001, *η*_*p*_^*2*^ = .87, and block, *F*(5, 85) = 13.95, *p* < .001, *η*_*p*_^*2*^ = .45. Once again, the former likely reflects the progressive training being easier overall than the constantly hard training regimen. The latter is likely driven by the large decrease in sensitivity over the course of training due to changes in stimulus contrast difficulty from block to block in the progressive condition. A significant range x block interaction, *F*(5, 85) = 39.77, p < .001, *η*_*p*_^*2*^ = .70, was also found. This likely stems from *A’s* for the two ranges converging over the course of the training period. These effects were expected and are unsurprising. Manipulations to stimulus similarity are well known to impact discriminability. We turn next to analyses of the test data to assess our hypotheses.

### Testing

**Fig 5:**
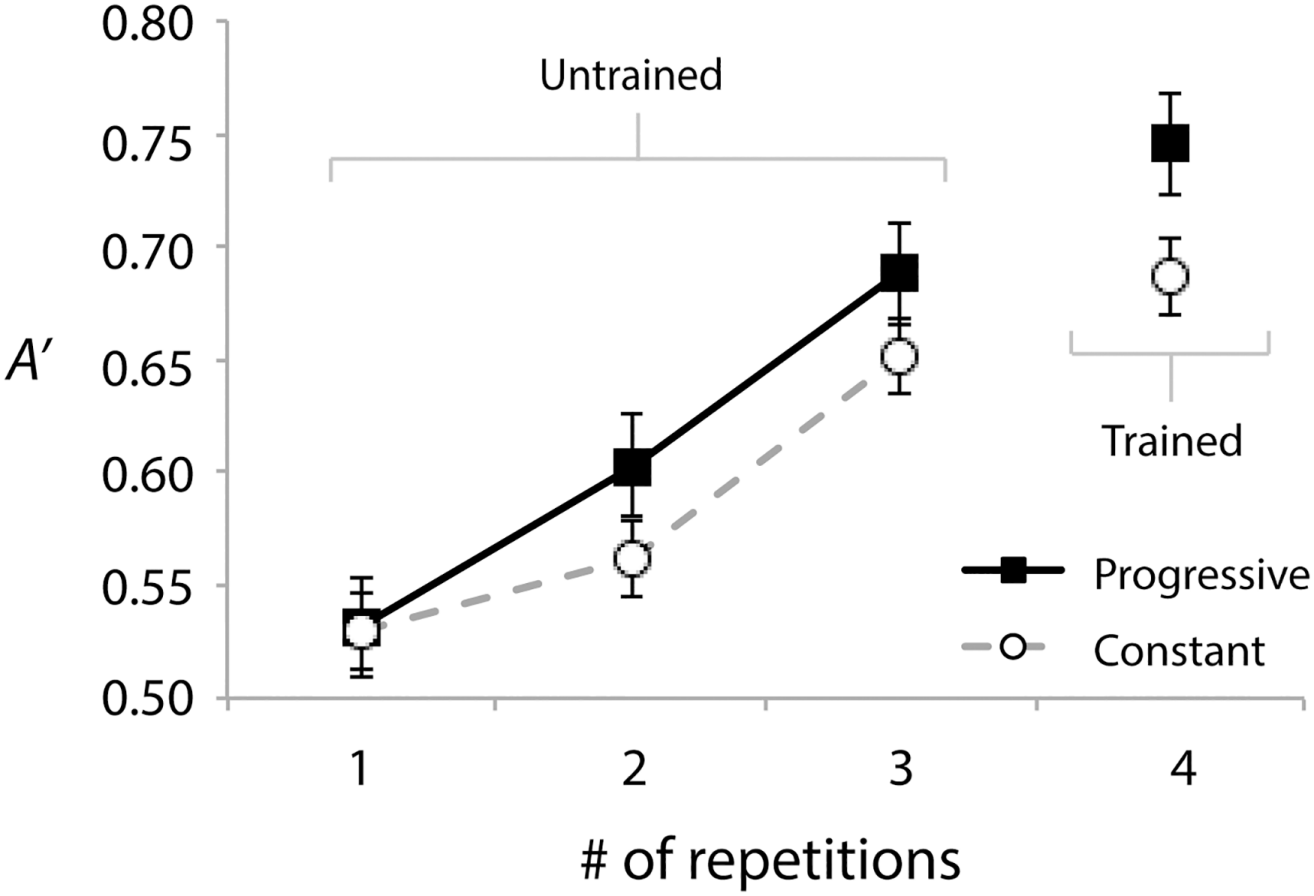
Test performances (*A’*) for the progressive (filled squares) and constant (open circles) trained frequency ranges (collapsed across counterbalance conditions) at each level of the repetition factor. Error bars show within-subject standard errors of the mean [57]. Trained and untrained numbers of repetitions are labeled.

Fig 5 shows test *A’* for the progressive and constant frequency ranges for each level of the repetition factor (1–4 repetitions). Qualitatively, it appears as though test performance is better in the progressively trained frequency range for every level of repetition, with the possible exception of stimuli containing a single sweep. A 2 (range: progressive or constant) x 4 (repetitions) repeated-measures ANOVA revealed a significant main effect of range, *F*(1, 17) = 7.72, *p* = .013, *η*_*p*_^*2*^ = .31, supporting better performance for the progressively trained range. There was also a significant main effect of repetition, *F*(3, 51) = 27.31, *p* < .001, *η*_*p*_^*2*^ = .62. A significant linear trend analysis was in support of FM sweep trains with more repetitions being easier to discriminate, *F*(1, 17) = 44.68, *p* < .001, *η*_*p*_^*2*^ = .72. The range x repetitions interaction was not significant, *F*<2.

## Discussion

Experiment 2 replicated the Experiment 1 finding that the progressively trained range showed enhanced performance post-training. Further, the progressive advantage was observable in an untrained 2i-2afc task, suggesting that the processes involved in learning are task-general rather than task-specific. The progressive advantage was also not stimulus specific. That is, the effect occurred for both trained and untrained sounds that contained less FM sweep repetitions compared to training.

## General discussion

The notion that focusing attention on a relevant perceptual dimension explains perceptual learning and easy-to-hard effects has been around for over a century [1,9,11]. In early writing on the topic, James [1] reported that most scientists/philosophers of the time had dismissed perceptual learning as a topic of study, assuming that the theoretical mechanisms were determined on the basis that: “what we attend to we perceive more minutely”. Unsatisfied with this as the sole explanation for perceptual learning, James offered a theory in which sensory impressions are discriminated based upon their associations with memories of past events, which could also account for easy-to-hard effects. Later, in a classic easy-to-hard effect demonstration, Lawrence [9] found that rats given 30 initial easy trials performed better than rats trained in a constantly hard regimen to discriminate stimulus brightness. He concluded that dimensional discovery should play some role in learning theory in addition to associative mechanisms (also see [58]). Simulation work later revealed that such effects could be accounted for by both associative (e.g., [20, 47]) and non-associative [18–19] representation-based learning mechanisms without assuming dimensional discovery. Even so, attentional-spotlighting views have continued to be popular accounts of the easy-to-hard effect and perceptual learning.

Here, we tested the adequacy of attentional spotlighting alone to explain easy-to-hard effects using auditory tasks. In two experiments, easy-to-hard effects were found within individuals when stimulus contrasts with the same critical dimensions were assigned to different training regimens (progressive or constantly difficult). Additionally, a progressive advantage was found within individuals for untrained FM sweep train sounds that contained less perceptual information than the training set (Experiment 2). That is, a progressive training advantage was also found in the generalization of learning towards more difficult to discriminate novel stimuli. The easy-to-hard effects observed appear to be task-independent as they were found when testing the post-training ability to discriminate sounds in the trained categorization task (Experiment 1) and an untrained 2i-2afc psychophysical task (Experiment 2). It should also be noted that all individuals were given explicit instructions to discriminate sounds based on their speed, “Slow” and “Fast” labels for responses, and on-screen reminders that they should discriminate sounds using speed. That easy-to-hard effects were still observed despite these multiple sources of information about the relevant dimension runs counter to arguments from the attentional-spotlighting perspective that the establishment of dimensional relevance “erases” easy-to-hard effects [11].

The current work does not refute the claim that knowing what to pay attention to in perceptual discrimination tasks has beneficial effects. This has been well demonstrated. Listeners trained to discriminate sounds along the dimension of azimuthal auditory spatial separation with inter-aural level difference (ILD) cues generalize some of this learning to spatial discriminations using inter-aural time difference (ITD) cues [22]. That learning generalizes even though the acoustic features available for discrimination are different has been taken as evidence that ILD trained participants learn to pay attention to auditory space. Also, discrimination of stimuli along category relevant dimensions can benefit from category learning regardless of whether or not comparison stimuli are on opposite sides of the categorization boundary (e.g., [59]; although, see [25]). An attentional-spotlighting view provides a reasonable account for these findings and several other related studies (for review, see [21,60–62]). Part of the reason why attentional-spotlighting continues to be used as a primary explanation for perceptual improvements is that the focus of attention has such large and consistent effects. However, learning that can take place with essentially no perceptual experience (e.g., by telling a person what to pay attention to; cf. [11]) does not well characterize perceptual learning as typically defined–“an increase in the ability to extract information from the environment, as a result of experience and practice with stimulation coming from it” [42].

Determining which representation-based mechanisms contribute to easy-to-hard effects, and perceptual learning more generally, has proven to be difficult [16–17,19,29]. Given that non-associative models like Saksida’s SOM based model [18–19] do not rely on reinforcement learning, one prediction they make is that a progressive advantage should be observed even under mere-exposure conditions. A few behavioral studies have tested this prediction, with recent research finding such an effect (e.g., [6, 63], although see [47,64]). Another prediction from non-associative models, but not necessarily from associative models, is that progressive training should benefit acuity for a trained stimulus on a dimension irrelevant for making the trained discrimination. Both the auditory [45] and visual domains [38] have shown task-irrelevant perceptual learning (although, see [58]). Non-associative models could potentially explain easy-to-hard effects in task-irrelevant perceptual learning if expansion of a stimulus’s representation in representational space (cf., [34]) aids in discriminating that stimulus from others that differ on more than the trained dimension. To our knowledge, predictions regarding the effects of stimulus sequencing in such a case have not yet been explicitly tested.

Whatever representation-based account fits the experimental data best will likely need to be merged with some model of attention in order to account for all circumstances under which easy-to-hard effects manifest. One possible framework for this is reverse-hierarchy theory (RHT). RHT proposes that easy discriminations recruit high-level cortical areas that focus processing on salient features [49,60]. As difficulty increases, lower cortical areas that form representations with higher resolution for those features are subsequently accessed by attention. Changes in stimulus representations and representational outputs play no role in easy-to-hard effects in this framework. Rather, effects stem from finding the appropriate existing representations for making distinctions. Aside from differences in the terms “appropriate dimension” and “appropriate representation”, the reasoning behind the RHT and dimensional discovery views is similar—easy trials help direct the “spotlight” of attention, which facilitates discrimination of difficult contrasts. RHT in its current form is also unlikely to account for the progressive advantage seen here since access to the representational level suitable for making distinctions should be the same for both progressively and constantly trained ranges within an individual. However, RHT theorists have acknowledged the need for representation-based learning within and/or between hierarchical levels to explain varieties of perceptual learning data (e.g., reweighting of features from low-levels to high-levels; [60]). Potentially, a model that specifies a representation-based learning process within RHT could account for easy-to-hard effects that appear to be driven by both attentional-spotlighting and representation-based learning mechanisms.

### Caveats and further considerations

This work was not designed to test all of the procedural and stimulus conditions under which easy-to-hard effects manifest. Because of this, it is necessary to consider how alternative conclusions reached by others may relate to methodological differences. Perhaps the most salient difference between our work and most of the other studies of the easy-to-hard effect is that we use auditory stimuli. Those arguing for attentional spotlighting have primarily used visual stimuli [5,11,49], even though similar processes are assumed to occur in the auditory system as well [11, 60]. The processing in the visual and auditory systems is distinct in several ways. This includes differences in the processing of temporal features, integration across dimensions, and occlusion/masking [60, 65]. Some claim these differences lead to qualitatively different learning effects [65]. A visual perceptual learning study analogous to ours may not produce similar results. In addition, we have also only tested easy-to-hard effects in tasks in which acoustic frequency modulation is relevant. Many other acoustic and non-auditory sensory dimensions remain to be tested.

Another difference between our methods and those of studies advocating for an attentional-spotlighting perspective lies in the nature of the tasks being trained. Whereas we attempted to minimize procedural learning (e.g., learning what dimensions determine category membership), others have used tasks wherein procedural learning is necessary. For instance, in Pashler and Mozer’s [11] experiments, the largest easy-to-hard effect was found in a categorization task with artificial face-like stimuli containing variations in eye size, the presence of a nose, brightness, and horn height. Only horn height was relevant. Participants were not told which feature was relevant and had to learn this through trial and error. In another categorization study, Spiering and Ashby [66] trained participants to categorize visual stimuli in a manner that required the use of spatial frequency and orientation dimensions. In contrast to our previous work, they found that participants trained with an anti-progressive regimen actually performed better than progressively trained participants. Presumably this was because hard-to-easy sequencing discouraged an inappropriate single-dimensional rule-based categorization strategy (i.e., using only one dimension). Learning-related changes to performance in these cases were likely related to discovering what to do rather than to any changes in perceptual acuity, especially since the tasks involved scenarios in which participants were not informed about the relevant dimensions. Though our data show different patterns of improvement and generalization, they are at odds with these earlier visual studies only in the sense that our findings demonstrate a need to consider learning processes beyond the discovery of task-appropriate strategies. Variations in the sequential structure of training regimens may affect procedural learning in ways that differ from their effects on perceptual learning.

### Practical and applied relevance

It is important to consider incremental processes of perceptual learning in the design of training regimens meant to address real-world issues of perception. Partly, this is because the involvement of these processes predicts consequences that are not considered or predicted by attentional-spotlighting. For instance, increased differentiation of perceptual representations in the brain may help individuals more flexibly use those representations in other cognitive tasks [67], allowing for the generalization of perceptual skills in a way not predicted by attentional spotlighting. Relatedly, better representational quality may reduce the need for one to utilize domain-general cortical networks involved in cognitive-control for determining what he or she is hearing or seeing (e.g., [68]). Learning could thus free up those resources for use in other tasks (e.g., encoding that information into long-term memory). Attentional spotlighting views instead propose that learning only involves correctly engaging cognitive control processes (e.g., selective attention). Another applied prediction from some representation-based learning models (e.g., [18–19]) is that exposure to stimuli should be beneficial even if task-relevant dimensions are not allocated attention. Potentially, such exposure could be used to bootstrap explicit perceptual training (cf. [69–70]). If designers of perceptual training regimens focus only on optimizing the “spotlight” of attention, they are unlikely to maximize their training procedures to fully utilize perceptual learning.

Collectively, past studies of the easy-to-hard effect along with the current results suggest that both attentional-spotlighting and basic incremental representation-based learning processes can be important in perceptual learning. Developers of training programs meant to reduce an individual’s perceptual or cognitive problems (e.g., language-related deficits; [71]), or to enhance performance in some perceptual task of interest (e.g., music perception; [72]; speech perception; [54]), should consider maximizing both types of learning. Programs that focus on either dimensional highlighting (e.g., [72–74]), or incremental learning processes (e.g., [75]), may not be as beneficial as programs that focus on both (e.g., [71,76]).

It is also important to note that the effects of sequencing that should be considered extend beyond the sequencing of discrimination difficulty. For instance, interleaved (e.g., A, B, A, B, A, B) and blocked (e.g., A, A, A, B, B, B) exposure to categories can have different effects on learning outcomes. Typically, interleaving categories throughout the training period leads to better performance (for review, see [77]). However, it may be that interleaved and blocked category training regimens have different effects depending upon perceptual similarities within a category. Hammer et al. [78] propose that blocked training should benefit performance when within category similarity is low (e.g., songbirds having different colors and shapes) because it will help a learner ignore category irrelevant information (e.g. color). In contrast, when within category similarity is high, the opposite should be true. That is, interleaving categories across trials will help the learners determine the features that make those categories distinct. The stimulus sets in our tasks conform to the latter circumstance. Progressive sequencing could have different effects in a categorization task if within-category similarity is low. Type of categorization task (e.g., ruled-based vs. information integration based) may also interact with easy-to-hard effects [66]. Hence, whether or not easy-to-hard benefits are obtained likely depend on the type of task being trained and the processes involved in learning (e.g., strategic rule-based learning vs. implicit long-term memory based learning). This may be important to consider for clinical populations known to use atypical categorization strategies (e.g., [79–80]).

Currently, decisions about when to increase or decrease difficulty, and the time to spend on various levels of difficulty within a task, are often determined by trial-and-error, self-reports of learners, or assumptions about what should work best. Potentially, learning could be simulated under a variety of training regimens using models that contain both incremental learning and attentional-spotlighting components. A subset of those training regimens leading to the best learning and generalization could then be tested in behavioral work. Similar methods have been used successfully in memory research (for review, see [81]), and could make it possible to design empirically validated training regimens, without an exhaustive corpus of behavioral studies. This work could also be informative in establishing training procedures that limit “worsening” in generalization (i.e., when training hurts performance with novel stimuli; [22,45,82–84]), or that optimally benefits learning for specific types of perceptual input.

## Conclusions

Although attentional spotlighting can in some cases be useful for learning to make fine perceptual distinctions, it alone is not a sufficient explanation of easy-to-hard effects. Attentional-spotlighting accounts incorrectly predict that easy-to-hard sequencing should aid discrimination performance all along the discrimination relevant dimension. They also incorrectly predict that when a participant’s attention is explicitly and repeatedly drawn to relevant dimensions early in training (e.g., by the presentation of easy contrasts in one range of that dimension), then he or she should show no within-subject benefits of progressive training (e.g., [11]). In contrast to the attentional-spotlighting explanation of easy-to-hard effects, proposed representational modification/reweighting learning mechanisms (e.g., [16,19,30]) are able to account for the specificity of easy-to-hard effects to trained sounds and the presence of an easy-to-hard effect when relevant dimensions are clearly revealed. Future theoretical and applied work may benefit from consideration of how multiple processes contribute, and possibly interact, to modify perceptual acuity.

## Supporting information

S1 File Supplementary experiment and analysis. This file describes a supplementary experiment demonstrating similar discriminability of rates in the ‘low’ and ‘high’ frequency ranges. It also presents an alternative analysis of data from Experiments 1 and 2 that includes counterbalance condition as a factor.
(DOCX)

S2 File Experiment 1 data files (Exp1data.zip). The files within are raw data files from Experiment 1. The readMe.txt file describes how to interpret the raw data. Due to restrictions placed on access to data collected at the U.S. Air Force Research Laboratory, access to Experiment 2 data must be requested through MGW.
(ZIP)
