# Supplementary material for: Benefits of fading in perceptual learning are driven by more than dimensional attention
Source: PLoS One. 2017 Jul 19;12(7):e0180959. doi: 10.1371/journal.pone.0180959 (PMC5516993; doi:10.1371/journal.pone.0180959)
Supplement: S1 File — This file describes a supplementary experiment demonstrating similar discriminability of rates in the ‘low’ and ‘high’ frequency ranges. It also presents an alternative analysis of data from Experiments 1 and 2 that includes counterbalance condition as a factor. (DOCX) [file pone.0180959.s001.docx]

**Supplemental Experiment**

A supplemental experiment was conducted to determine whether or not FM sweep trains spanning frequencies in a low (300 – 600 Hz) and high (3000 – 6000 Hz) frequency range yield comparable performances for participants discriminating sounds using the dimension of FM rate.

**Methods**

**Participants.** Twenty individuals at the U.S. Air Force Research Laboratory, Wright-Patterson Air Force Base, OH, were either paid to participate, or served as unpaid volunteers. All signed a U.S. Air Force Institutional Review Board approved informed consent document. All individuals had prior experience participating in psychoacoustic studies. Eighteen individuals went on to complete Experiment 2 after participating in this experiment.

**Stimuli and apparatus.** Sweep trains contained 1, 2, 3, or 4 sweeps each. All sweep trains contained upward sweeps that spanned frequencies from 300 – 600 Hz (low) or 3000 – 6000 Hz (high). FM rates of 6.3 and 6.8 octaves per second were used.

Experimental procedures and data acquisition were performed using MATLAB R2013a (Natick, MA). Participants made responses via a computer keyboard. Sounds were presented over Telephonics TDH-39P headphones (Farmingdale, NY) in an Acoustic Systems sound booth (Occupational Health Dynamics, Hoover, AL).

**Design & Procedures.** On each trial two FM sweep train stimuli were presented, separated by 500 ms. One stimulus was “Fast” (6.8 octaves per second) and one was “Slow” (6.3 octaves per second). Listeners’ task was to indicate which was faster. Repetitions in FM sweep stimuli (1 - 4) varied from trial to trial, but were identical for the two stimuli presented within a trial. There were 4 blocks in each test with 48 trials in each. The order of fast and slow sounds within a trial (i.e., whether the faster sound occurred first or second) was counterbalanced. Trials were completely randomized within a block. No feedback was presented. There was no response deadline.

**Results & Discussion**

The nonparametric signal detection *A*’ measure was computed using methods outlined by Macmillan and Creelman (1991) to assess discriminability. Figure 1 shows *A’* for the low (300 – 600 Hz) and the high (3000 – 6000 Hz) frequency ranges for each level of the repetition factor (1 – 4). For 1, 2, and 4 repetitions, *A’* was basically indistinguishable for the low and high frequency ranges. There was a qualitative difference for sweep trains containing 3 repetitions, trending for better performance for the high frequency range. However, a 2 (frequency range) x 4 (repetition) repeated-measures ANOVA failed to reveal any significant main effect of frequency range, *F*<2. The frequency range x repetition interaction also failed to reach significance, *F*(3,57) = 2.14, *p* =.11, *η_p_^2^*=.10.

As expected there was a significant main effect of repetition, *F*(3, 57) = 11.84, *p*<.001, *η_p_^2^*=.38, supporting easier discrimination of rates with greater numbers of repetitions. All together, the results from this preliminary experiment suggest that frequency range should have minimal impacts on sequencing effects observable by using different training regimens in different frequency ranges.

***A’***

**Repetitions**

Figure S1. Discriminability (*A’*) for each frequency range and level of the repetition factor (1-4 repetitions). Error bars show within-subject standard errors of the mean ($\sqrt{MSE/n}$).

**Experiment 1 Supplemental Analysis**

See Experiment 1 in the paper for details on the experimental methods. Accuracy data (*A’*) that includes counterbalance condition as a factor is shown in Figure S2. A 2 (counterbalance condition: high-progressive, low-progressive) x 2 (range: progressive, constant) mixed-model ANOVA found a significant main effect of range, *F*(1,18) = 5.10, *p*=.037, *η_p_^2^*=.22, corroborating the progressive advantage reported in the original analysis. The main effect of counterbalance condition was marginally significant, *F*(1,18) = 4.13, *p* = .057, *η_p_^2^*=.19, trending such that the high-progressive counterbalance condition outperformed the low-progressive counterbalance condition. This is possibly related to the random assignment process resulting in better listeners in the low-progressive condition. The interaction was not significant, *F*(1,18) = 2.67, *p* = .12, *η_p_^2^*=.13.


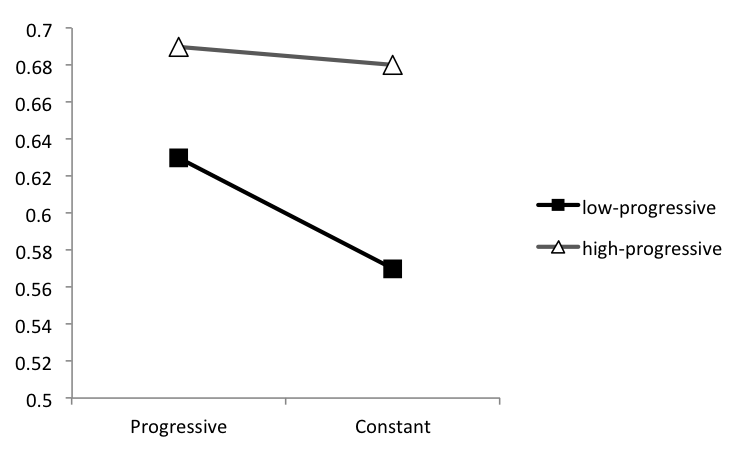


*A’*

Figure S2. Accuracy (*A’*) for the progressive and constant trained frequency ranges for each counterbalance condition. Low-progressive is the condition in which the ‘low’ frequency range received progressive training. High-progressive is the condition for which the ‘high’ frequency range received progressive training.

**Experiment 2 Supplemental Analysis**

See Experiment 2 in the paper for details on the experimental methods. Accuracy data (*A’*) that includes counterbalance condition as a factor is shown in Figure S3. A 2 (counterbalance condition: high-progressive, low-progressive) x 2 (range: progressive, constant) x 4 (repetitions: 1, 2, 3, or 4) mixed-model ANOVA found a significant main effect of range, *F*(1,16) = 7.65, *p*=.014, *η_p_^2^*=.32, corroborating the progressive advantage reported in the original analysis. The main effect of repetitions was also significant, *F*(3,48) = 25.78, *p* <.001, *η_p_^2^*=.62, as in the original analysis. No other main effects or interactions were significant.


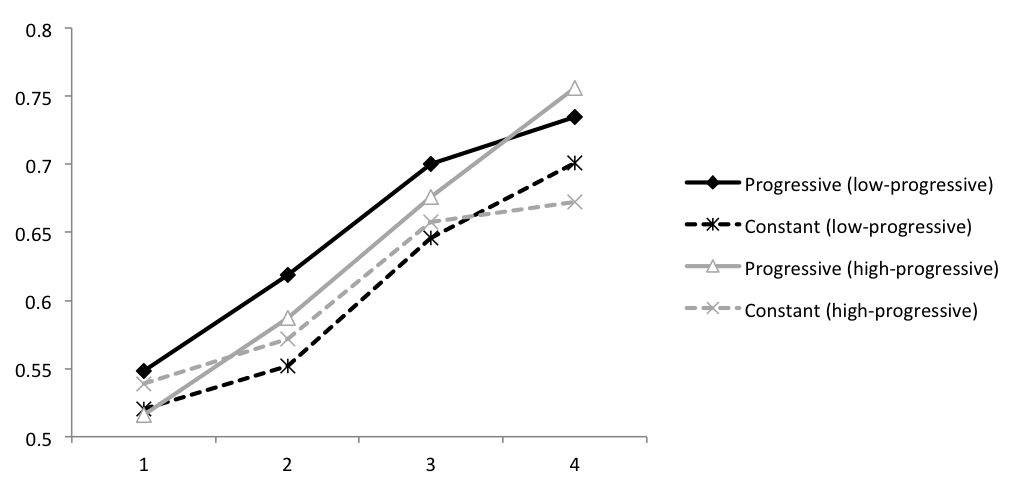


*A’*

Repetitions

Figure S3. Accuracy (*A’*) for the progressive and constant trained frequency ranges for each counterbalance condition. Low-progressive is the condition in which the ‘low’ frequency range received progressive training. High-progressive is the condition for which the ‘high’ frequency range received progressive training.
