# Supplementary material for: Benefits of fading in perceptual learning are driven by more than dimensional attention
Source: PLoS One. 2017 Jul 19;12(7):e0180959. doi: 10.1371/journal.pone.0180959 (PMC5516993; doi:10.1371/journal.pone.0180959)
Supplement: S2 File — The files within are raw data files from Experiment 1. The readMe.txt file describes how to interpret the raw data. Due to restrictions placed on access to data collected at the U.S. Air Force Research Laboratory, access to Experiment 2 data must be requested through MGW. (ZIP) [file pone.0180959.s002.zip › readMe.rtf]

The documents in this folder are raw data files for Experiment 1 of Wisniewski et al. (2017; PLoS ONE).  This file describes the contents.***************************************.azk filesRaw data output.  Hard_v_Prog files are files in which the low-pitch range received constantly hard training and the high pitch range received easy-to-hard training.  The opposite is true for the Prog_v_Hard files.  The “COMPX” ends to these file names refers to the lab computer the files were obtained from (We used 3 different computers with the same setup).Each individual has a header line in these files that states the date run and the 3 digit subject ID (e.g., 201).  There are two columns for data, each row is a different trial.  The first column gives that trial’s 4-digit code (ABCD).  Here, A - block number, B - pitch range (1=low, 2=high), C = unvaried variable of loudness (no meaning for this study), and D = rate code from 1-8 (from slow to fast; see paper methods).  The second column labels “RT” is the response time and correctness column.  Positive values are correct trials, negative values are incorrect trials.  The number corresponds to the response time since beginning of sound playback.  Note that response time accuracy is variable in our setup and there are several trials which have warnings of response time errors.  We do not formally analyze RTs for this reason.  Any analyses of RT should be made with caution.Note: subID 211 in Hard_v_ProgCOMP3.azk and subID 214 in Hard_v_ProgCOMP3.azk were collected after the desired n had already been reached and made subject counts in the counterbalance conditions was not equal.  This was an error of our research assistant.  These subjects were not included in analyses, but remain in the raw files for full presentation of data.
